# Effect of dietary polyunsaturated fatty acid and antioxidant supplementation on the transcriptional level of genes involved in lipid and energy metabolism in swine

**DOI:** 10.1371/journal.pone.0204869

**Published:** 2018-10-04

**Authors:** Marika Vitali, Corrado Dimauro, Rubina Sirri, Martina Zappaterra, Paolo Zambonelli, Elisabetta Manca, Dalal Sami, Domenico Pietro Lo Fiego, Roberta Davoli

**Affiliations:** 1 Interdepartmental Centre of Industrial Agrifood Research (CIRI- AGRO) University of Bologna, Cesena, Italy; 2 Department of Agronomy, University of Sassari, Sassari, Italy; 3 Department of Agricultural and Food Sciences (DISTAL), University of Bologna, Bologna, Italy; 4 Department of Life Sciences, University of Modena and Reggio-Emilia, Reggio Emilia, Italy; 5 Interdepartmental Research Centre for Agri-Food Biological Resources Improvement and Valorisation (BIOGEST-SITEIA), University of Modena and Reggio Emilia, Reggio Emilia, Italy; University of Illinois, UNITED STATES

## Abstract

Porcine fat traits depend mostly on the interaction between nutritional and genetic factors. However, the pathways and biological processes influenced by this interaction are still poorly known in pigs, although they can have a huge impact on meat quality traits. The present research provides new knowledge insight into the effect of four diets (D1 = standard diet; D2 = linseed supplementation; D3 = linseed, vitamin E and selenium supplementation; D4 = linseed and plant-derived polyphenols supplementation) on the expression of 24 candidate genes selected for their role in lipid and energy metabolism. The data indicated that 10 out of 24 genes were differentially expressed among diets, namely *ACACA*, *ADIPOQ*, *ADIPOR1*, *CHREBP* (*MLXPL*), *ELOVL6*, *FASN*, *G6PD*, *PLIN2*, *RXRA* and *SCD*. Results from the univariate analysis displayed an increased expression of *ACACA*, *ADIPOQ*, *ADIPOR1*, *CHREBP*, *ELOVL6*, *FASN*, *PLIN2*, *RXRA* and *SCD* in D4 compared to D2. Similarly, *ACACA*, *ADIPOQ*, *ADIPOR1*, *ELOVL6* and *SCD* were highly expressed in D4 compared to D3, while no differences were observed in D2-D3 comparison. Moreover, an increased expression of *G6PD* and *ELOVL6* genes in D4 compared to D1 was observed. Results from the multivariate analysis confirmed that D2 was not different from D3 and that *ACACA*, *SCD* and *FASN* expression made D4 different from D2 and D3. Comparing D4 and D1, the expression levels of *ELOVL6* and *ACACA* were the most influenced. This research provides evidence that the addition of both *n*-3 PUFA and polyphenols, derived from linseed, grape-skin and oregano supplementation in the diets, stimulates the expression of genes involved in lipogenesis and in oxidative processes. Results evidenced a greater effect on gene expression of the diet added with both plant extracts and *n*-3 PUFA, resulting in an increased expression of genes coding for fatty acid synthesis, desaturation and elongation in pig *Longissimus thoracis* muscle.

## Introduction

The phenotypic variability of porcine fat traits is regulated by many environmental and genetic factors, and several studies have demonstrated that both diet and genotype are the main factors influencing intramuscular fatty acid (FA) composition in all the animal species [1–3]. However, as the influence of diet on biological processes and pathways is poorly known [4–6], identification of genes involved in lipid metabolism and their relationship with diet is of main interest for meat quality research purposes in pigs. Many studies reported the importance of identifying the effect of lipogenic genes expression, in order to improve the knowledge on biological processes and metabolic pathways influencing muscle fat deposition to provide new insight into carcass adiposity [7,8]. In literature, several studies dealt with the effects of *n-*3 and *n-*6 polyunsaturated FA (PUFA) diet supplementation on lipid metabolism in skeletal muscle, adipose and liver tissues. These researches focused on the effect that different diets may have on the expression of genes involved in PUFA synthesis or in the regulation of FA composition [9–14]. However, the effect of PUFA dietary supplementation on the regulation of lipid metabolism through the modification of gene expression in swine is still poorly understood. De Tonnac et al. [12] found that the intake of docosahexaenoic acid (DHA) down-regulated the transcription of genes involved in fatty acid (FA) metabolism regulation such as *FADS2* and *SREBP1* in the liver and *DECR2* in the *Longissimus thoracis* muscle of growing-finishing cross-breed pigs. In a study on the transcription profile of porcine *Gluteus medius* muscle, Ogłuszka et al. [14], showed that a diet supplemented with *n-*3 and *n-*6 PUFA led to the down-regulation of genes coding for apolipoproteins. Tous et al. [10] reported that dietary conjugated linoleic acid (CLA) can affect the expression of both porcine lipogenic and regulatory genes including *PPARA*, *PPARG*, *FASN*, *SREBF1*, *ACACA*, *LPL*, *D6D*, *SCD* in a tissue-specific manner. Some studies have described that supplementing the diet with antioxidants or polyphenols can also influence nutrient digestibility, gut microbiota, expression of pro-inflammatory genes and meat quality traits in pigs [15–19]. Overall, the effects of dietary supplementation on gene expression are mostly unknown. In particular, the effect of a diet enriched with both *n-*3 PUFA and antioxidants or polyphenols on the expression of genes involved in lipid metabolism of pig muscle has not been studied by other Authors until now.

The aim of the present research is to investigate, for the first time in pig skeletal muscle, the influence of dietary functional bioactive compounds on gene expression, using different statistical approaches. The expression level of a set of selected genes involved in lipid and energy metabolism and in fat deposition was tested in the *Longissimus thoracis* muscle samples of Italian Large White (ILW) pigs. Pigs were fed three different diets supplemented with extruded linseed (which is a natural source of *n-*3 PUFA), vitamin E and selenium and plant extract (as a source of polyphenols). Furthermore, in this research, two different statistical approaches are proposed. Besides the univariate analysis (ANOVA), the multivariate canonical discriminant analysis (CDA) was performed to account for the correlation among the genes considered and to enlighten gene relationships, in order to identify processes that can be influenced by the diets.

## Material and methods

### Ethics approval

All the experimental procedures performed in this study were in accomplishment with the national legislation and did not require special animal care authorizations according to the decision of the welfare committee of Consiglio per la Ricerca in agricoltura e l’analisi dell’economia agraria (CREA) taken the 14 September 2016 (Verbale 2) according to the Italian legislation, D.Lgs 4 Marzo 2014 n. 26 art. 2 punto F.

### Animals and sampling

A total of 48 ILW pigs (23 gilts and 25 barrows) were used for this study. The pigs were selected from a progeny of 258 piglets derived from 21 sows and 3 boars provided by the Italian National Association of Pig Breeders (ANAS, Roma, Italy). After weaning, the selected pigs were divided into 4 groups of 12 pigs, balanced for weight, father and sex. After weaning all pigs were fed a standard diet until the starting of the trial. Then the trial started when pigs were 79.9 kg ± 5.8 kg until they reached 150.5± 9.9 kg. During this period, the pigs were fed four experimental diets: a standard diet for growing-finishing pig (D1); a diet enriched with extruded linseed (5% of feed) (D2); a diet enriched with extruded linseed, vitamin E and selenium (250 and 0.31 mg per kg of feed respectively) (D3); a diet enriched with extruded linseed and plant extract from grape-skin (3 g/kg feed; Enocianina Fornaciari s.n.c., Reggio Emilia, Italy) and oregano (2 g/kg feed; Phenbiox Srl, Bologna, Italy) as polyphenols source (total polyphenols added 37.6 mg per kg of feed) (D4). The chemical composition of extruded linseed was characterized as follow: moisture (8%), crude fiber (25.0%), crude protein (20.2%), crude lipids (29.6%), and ashes (3.0%). Fatty acid composition had 54.7% of α-Linolenic acid of total fatty acids. The n-3 PUFA content (g per 100 g of total fatty acids) was mainly constituted of α-Linolenic acid and it was 5.2% in the control diet (D1) and 25.4% in D2, D3 and D4. The analytical total content of polyphenols contained in plant extracts was 10.4 g/L for grape-skin extract and 3.9 g/L for oregano extract. During the first period, which ranges from an average weight of 79.9 ± 5.8 kg to 113.4 ± 10.6 kg, the amount of the supplied meal was set as 7.5% of the metabolic live weight (1^st^ on Table 1). During the finishing period, from 113.4 ± 10.6 kg to the slaughter (average weight of 150.5 ± 9.9 kg) the amount of the supplied meal was set as 8.5% of the metabolic live weight (2^nd^ on Table 1). The detailed composition of the four experimental diets and nutritional contents are reported in Table 1. During the rearing period, the weight of each pig was recorded at birth, at the starting of the trial (*i*.*e*. before the experimental diets were administered), in mid-trial and one day before slaughter.

**Table 1:** Feed components and proximate composition of the experimental diets on an as-fed basis.

		D1		D2		D3		D4	
*Ingredients*		1^st^	2^nd^	1^st^	2^nd^	1^st^	2^nd^	1^st^	2^nd^
**Extruded linseed**	%	0.00	0.00	5.00	5.00	5.00	5.00	5.00	5.00
**Barley meal**	%	85.50	91.00	80.50	86.60	80.30	86.40	80.50	86.60
**Soya bean meal**	%	11.00	5.50	11.00	5.00	11.00	5.00	11.00	5.00
**L-Lysine**	%	0.31	0.29	0.30	0.29	0.30	0.29	0.30	0.29
**DL-Methionine**	%	0.06	0.04	0.06	0.03	0.06	0.03	0.06	0.03
**L-Threonine**	%	0.05	0.04	0.05	0.03	0.05	0.03	0.05	0.03
**Calcium carbonate**	%	1.18	1.13	1.19	1.15	0.89	0.85	1.19	1.15
**Dicalcium phosphate**	%	1.00	1.10	1.00	1.00	1.00	1.00	1.00	1.00
**Salt (NaCl)**	%	0.40	0.40	0.40	0.40	0.40	0.40	0.40	0.40
**Vitamin/mineral pre-mix**^1^	%	0.50	0.50	0.50	0.50	0.50	0.50	0.50	0.50
**Vitamin E and Selenium pre-mix**^2^	%	0.00	0.00	0.00	0.00	0.50	0.50	0.00	0.00
**Plant extracts (Grape-skin + oregano)**	g *per* kg of feed	0.00	0.00	0.00	0.00	0.00	0.00	3.00+2.00	3.00+2.00
***Proximate composition***									
**Digestible energy**	kcal/kg	3189	3168	3255	3235	3248	3228	3255	3235
**Crude protein**	%	14.89	11.31	15.39	11.73	15.37	11.71	15.39	11.73
**Crude fat**	%	1.75	1.74	3.58	3.58	3.58	3.58	3.58	3.58
**Crude fiber**	%	4.33	4.2	4.62	4.48	4.61	4.47	4.62	4.48
**Ca**	%	0.80	0.79	0.82	0.79	0.82	0.79	0.82	0.79
**P**	%	0.54	0.54	0.55	0.53	0.55	0.53	0.55	0.53
***Fatty acid composition***	% (of total fatty acids)								
**C14:0**	%	0.47	0.39	0.25	0.21	0.25	0.22	0.26	0.22
**C16:0**	%	29.01	24.25	18.13	15.20	17.78	15.59	18.80	15.31
**C16:1**	%	0.49	0.34	0.17	0.15	0.17	0.17	0.02	0.15
**C18:0**	%	2.03	1.51	4.00	3.18	3.88	3.34	4.16	3.23
**C18:1 *n-*9**	%	14.92	13.50	20.60	18.12	20.24	18.45	21.29	18.26
**C18:2 *n-*6**	%	47.55	53.67	33.50	34.69	33.91	34.09	32.52	34.47
**C18:3 *n-*3**	%	4.77	5.70	22.83	28.02	23.25	27.73	22.38	27.95
**C20:1**	%	0.74	0.64	0.53	0.41	0.52	0.42	0.57	0.41

D1 = standard diet for growing-finishing pigs; D2 = standard diet supplemented with linseed (source of *n-*3 PUFA); D3 = standard diet supplemented with linseed, vitamin E and selenium (250 and 0.31 mg *per* kg of feed respectively; D4 = standard diet supplemented with linseed and plant extracts from grape-skin (3 g *per* kg of feed) and oregano (2 g *per* kg of feed) as polyphenols source (total polyphenols added 37.6 mg *per* kg of feed).

1^st^ = feed administered from an average weight of 79.9 kg to 113.4 kg; 2^nd^ = feed administered from an average weight of 113.4 kg to slaughter.

^1^Provided the following nutrients (*per* kg diet as-fed): Vitamin A 15,000 IU; Vitamin D3 2,000 IU; Vitamin E alpha-tocopheryl-acetate), 50 mg; Vitamin K, 2.5 mg; Vitamin B1, 2 mg; Vitamin B2 5 mg; Vitamin B5, 15 mg; Vitamin B6, 4 mg; Vitamin B12, 0.036 mg; Niacin, 25 mg; Folic acid, 1 mg; Biotin, 0.15 mg; Choline, 346 mg; Cu, 15 mg; Fe, 150 mg; Mn, 25 mg; Co, 0.4 mg; I, 1.5 mg; Zn, 100 mg; Se) 0.1 mg.

^2^Provided the following nutrients (*per* kg diet as-fed): Vitamin E (alpha-tocopheryl-acetate), 200 mg and Se, 0.21 mg.

At the end of the trial, the pigs were slaughtered in a commercial abattoir where the pigs were electrically stunned and bled in a lying position in agreement with the Council Regulation (EC) No 1099/2009 on the protection of animals at the time of the killing. All slaughter procedures were under the control of the Veterinary Service from the Italian Ministry of Health. The slaughter was performed in two batches, at an interval of 14 days. Each batch was composed by the half (6) of pigs per each experimental groups (6 pigs/group = 24 pigs/batch). Heaviest pigs were sent in the first batch. Because one barrow died before the ending of the trial (due to an abdominal hernia), D4 was composed of 5 pigs at the second batch.

At the end of the slaughter line and before the carcass cooling, two samples of the muscle *Longissimus thoracis* were taken from each pig and immediately frozen in liquid nitrogen. Samples were then stored at -80°C until RNA extraction.

### Gene expression analysis

Total RNA was extracted from 250 mg of a frozen sample of *Longissimus thoracis* muscle *per* each pig and homogenized in Trizol reagent (Invitrogen Corporation, Carlsbad, California), following the protocol described in Davoli et al. [20]. All samples were quantified through a NanoDrop 1000 Spectrophotometer (NanoDrop Technologies Inc., Wilmington, DE) at a wavelength of 260 nm. RNA purification from DNA was performed using the TURBO DNAse–free kit (Ambion, California, USA). RNA quality and genomic DNA presence were assessed by electrophoresis on Agarose gel. The synthesis of cDNA was performed from 1μg of RNA using the ImProm-II Reverse Transcription System (Promega Corporation, Italy), resulting in 20 μl of cDNA solution. Target genes intended for Reverse-Transcriptase quantitative PCR (RT-qPCR) were selected reviewing the literature according to their functional role in lipid metabolism, as summarized in Table 2 and S1 Table. Primers were designed using Primer3Plus (URL: http://www.bioinformatics.nl/cgi-bin/primer3plus/primer3plus.cgi), Primer-BLAST (URL: https://www.ncbi.nlm.nih.gov/tools/primer-blast/) and Operon Oligo analysis tool (URL: http://www.operon.com/tools/oligo-analysis-tool.aspx) online software, or were obtained from the literature. The complete list of target genes and references are shown in S1 Table. Then RT-qPCR was performed on Rotor-Gene 6000 (Qiagen, Hilden, Germany) using 5 μl of SYBR Premix ex Taq, 10 pmol of each Primer, 2 μl of cDNA template diluted to a concentration of 1:10 μl in nuclease-free water. cDNA was stored at -20°C in 10μl aliquots. RT-qPCR was performed using a two-step amplification with cycles constituted by a denaturation phase at 95°C for 5 seconds, followed by an annealing-extension step for 20 seconds at temperatures optimized for each primer couple (S2 Table). Three replicates for each sample (Table A in S1 File) were performed (2 replicates in a same RT-qPCR run and a third replicate in a separate run) and the variation coefficient was set at 0.2 as maximum level accepted. RT-qPCR runs were considered only if amplification efficiencies were high (slopes < -3.25 and R2 ≥ 0.99). These values were automatically calculated by Rotor Gene 6000 using dynamic tube normalization and noise slope correction.

**Table 2:** List of candidate genes considered in the present study and respective main functions.

Functional classification	Genes
β-oxidation	*ADIPOQ*, *ADIPOR1*, *ADIPOR2*, *ME1*, *PPARA*, *RXRA*
Fatty acid desaturation	*SCD*
Fatty acid elongation	*ELOVL6*
Glucose metabolism	*CHREBP*, *G6PD*, *PPP3CA*
Lipid storage	*PLIN2*, *PLIN3*, *PLIN5*
Lipogenesis	*ACACA*, *ACLY*, *CHREBP*, *FADS2*, *FASN*, *G6PD*, *SREBP1C*
Lipolysis	*ATGL*, *LIPE*, *LPL*, *MGLL*
Transcription factors	*CHREBP*, *LXRA*, *PPARA*, *PPP3CA*, *RXRA*, *SREBP1C*,

Nine reference genes (HKGs) were tested to assess the stability of their expression levels across samples: *ACTB*, *B2M*, *HPRT1*, *POLR2A*, *RPLP0*, *RPL32*, *RPS18*, *TBP*, *YWHAZ*. The selection of the best couple of HKGs to use was performed using geNorm [21] and NormFinder [22] software. Among them, *HPRT1* and *RPS18* were selected as the most stable couple in the 47 samples. This couple of genes was used as reference genes to quantify the expression of the target genes. For each individual, the relative quantification of a target gene was calculated dividing the mean obtained for the triplicate measurements of the target gene expression by the geometric mean of the two HKGs expressions (Table B in S1 File). The expression levels were calculated using the standard curve methods, according to Pfaffl [23]. Standard curves were obtained amplifying 12 progressive dilutions (from 10^9^ to 25 molecules/μl) of a cDNA sample at a known concentration, obtained by PCR. The absence of unspecific amplicons during RT-qPCR on Rotor-Gene 6000 was tested using the melting step after the cycling.

### Statistical analysis

*Univariate analysis*. The analysis was performed using R environment [24]. First, gene expression data were tested for normality distribution (Shapiro-Wilk test) and the CAR package [25] was used to normalize the data. Then data were analyzed using the analysis of variance (ANOVA), type III analysis of least square means (LSM), applying the following linear mixed model:

$$y_{ijklm}=\mu +SD_{i}+S_{j}+D_{k}+DA_{l}+\varepsilon_{m}$$

where y was the expression level of each gene; SD and S were the fixed effects of *slaughter day* (2 levels) and of *sire* (3 levels), respectively; D was the fixed effect of *diet* (4 levels); DA was the random effect of *dam* (21 levels) and ε the residual random error. Sex variable was removed from the model since in a previous analysis this factor did not show to affect any of the gene expression levels.

The random effects covariance was structured as **I**σ ^2^_dam_ and **I**σ ^2^_ε_, respectively, where **I** is an identity matrix and **I**σ ^2^_dam_ and **I**σ^2^_ε_ are sows and residual variance, respectively. The LSM was used to test differences between levels of fixed effects using the Tukey’s adjustment for pairwise comparisons.

*Multivariate analysis of covariance*. Multivariate analysis was performed using the SAS software (SAS Institute, Cary, NC, USA). Raw expression data (Table C in S1 File) were submitted to the canonical discriminant analysis (CDA), a dimension reduction technique able to perform both univariate and multivariate one-way analysis. CDA analysis was performed with the aim to identify genes whose expression was able to better discriminate the 4 diets. In general, if *k* is the number of the groups involved (the diets), the CDA derives *k-1* linear equations, called canonical functions (CAN), that are linear combinations of the original variables (gene expression data). The structure of a CAN is:

$$\mathrm{CAN}=c_{1}X_{1}+c_{2}X_{2}+\ldots \ldots +c_{n}X_{n},$$

Where X_*i*_ are the scores of the original variables and c_*i*_ are the canonical coefficients indicating the contribution of each variable in composing the CAN. Therefore, the higher the absolute value of c_*i*_, the higher the weight of the corresponding X_*i*_ in the CAN. The effective separation between dietary groups was tested by using the Mahalanobis’s distance and the corresponding Hotelling’s T-squared test [26]. Then, CDA analysis was applied only to significant differentially expressed (*P* < 0.05) genes and genes that showed a trend (*P* < 0.10) in the previous ANOVA analysis (DEGs). Then the stepwise discriminant analysis (SDA), a statistical technique specifically conceived to select the minimum subset of variables that better separate the groups, was applied. Lastly, a new run of CDA was performed using, as variables, only the genes selected through the SDA.

## Results

### Univariate analysis

**Fig 1:**
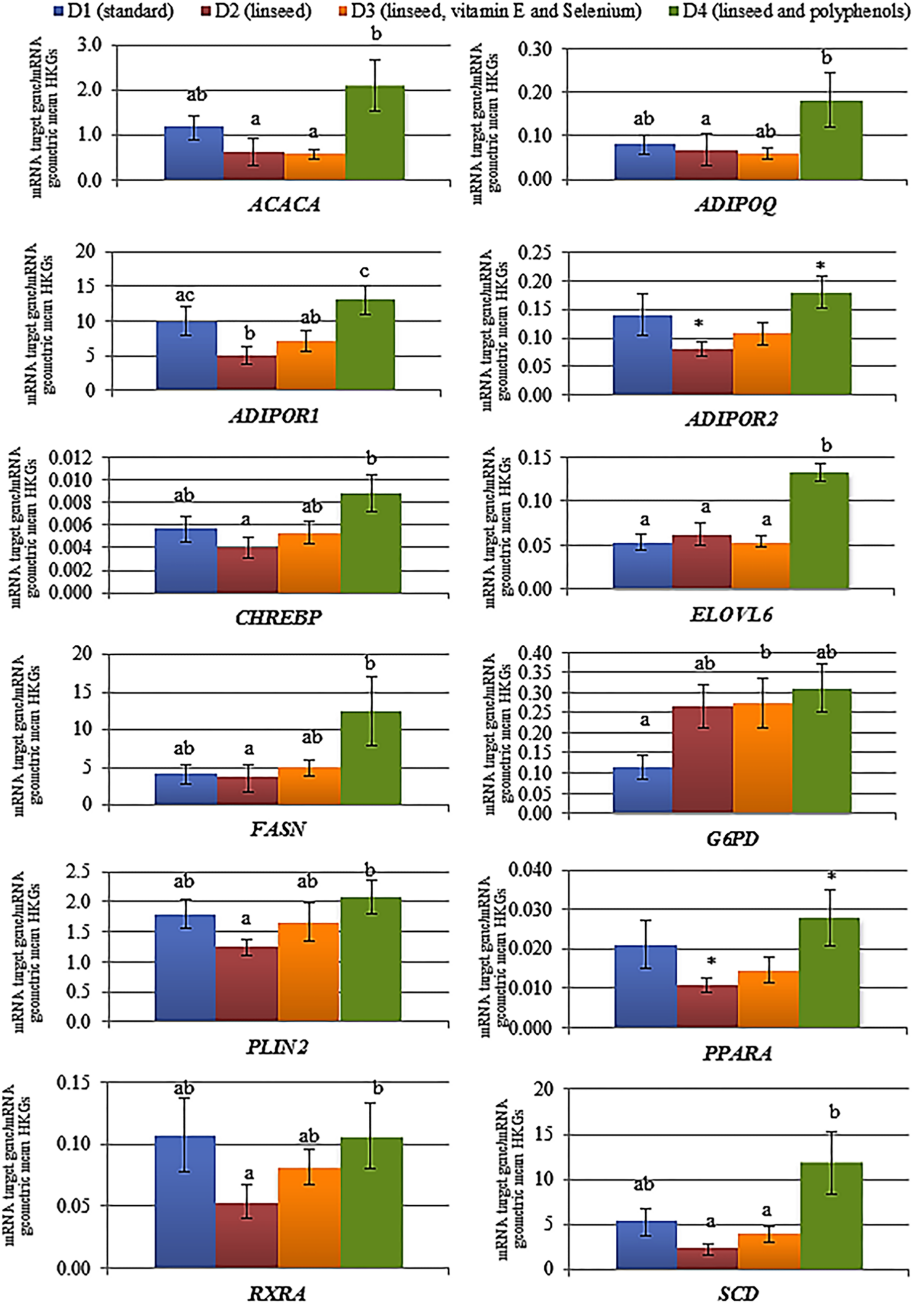
Gene expression levels in the experimental diets. Vertical bars in the histograms represent the standard error. Means within a row with different superscripts differ (a, b = *P* < 0.05) while means within an ascot present a trend (* = *P* < 0.10).

The results from univariate analysis indicated that ten (*ACACA*, *ADIPOQ*, *ADIPOR1*, *CHREBP*, *ELOVL6*, *FASN*, *G6PD*, *PLIN2*, *RXRA*, *SCD*) out of 24 genes presented a differential expression level among the experimental diets. Results of gene expression and pairwise comparisons between diets are graphically represented in Fig 1 and reported in S3 Table. Pairwise comparisons showed that most of the genes (9 out of 24) were significantly more expressed in D4 when compared to D2, namely *ACACA*, *ADIPOQ*, *ADIPOR1*, *CHREBP*, *ELOVL6*, *FASN*, *PLIN2*, *RXRA* and *SCD*. In particular, genes presenting the strongest differences between these two diets were *ADIPOR1* (*P* = 0.0008), *SCD* (*P* = 0.0011), *ACACA* (*P* = 0.0012) and *ELOVL6* (*P* = 0.0089). Furthermore, a trend towards significance was observed for *ADIPOR2* (*P* = 0.0663) and *PPARA* (*P* = 0.0881), which were more expressed in D4 (S3 Table).

When comparing D4 and D3, four genes (*ELOVL6*, *SCD*, *ACACA* and *ADIPOR1*) were significantly more expressed in D4 (*P* = 0.0128, *P* = 0.0326, *P* = 0.0055 and *P* = 0.032, respectively), while a trend was observed for *ADIPOQ* (*P* = 0.0544). In the D4-D1 comparison, *ELOVL6* and *G6PD* genes were more expressed in D4 than in D1 (*P* = 0.0048 and *P* = 0.0249. respectively). An increased expression of *G6PD* was also observed in D3 compared to D1 (*P* = 0.0427), while in D1-D2 comparison only *ADIPOR1* displayed an increased expression in D1 (*P* = 0.0363). Additionally, no significant differences (neither a trend) were found between D2 and D3 in all the target genes.

### Multivariate analysis

The CDA analysis was performed considering both DEGs (*ACACA*, *ADIPOQ*, *ADIPOR1*, *CHREBP*, *ELOVL6*, *FASN*, *G6PD*, *PLIN2*, *RXRA* and *SCD*) and genes presenting a trend (*PPARA* and *ADIPOR2*) based on univariate analysis results. Hotelling’s t-test *P-*values and Mahalanobis’s distances (Table 3) showed that the diets D2 and D3 have similar effect on gene expression as well as the diet D1 and D4 (*P* > 0.05); on the other hand, D2 and D3 were significantly different from D1 and D4 respectively (*P* < 0.01 in all comparisons). Distances between the diets are also graphically displayed in S1 Fig, where D1 and D4 are both located on the right part of the graph, while D2 and D3 are clustered in the left part.

**Table 3:** Hotelling’s T-test *P*-values (above diagonal) and Mahalanobis’s distances (below diagonal) between diets, calculated on the expression of a set of 12 genes selected because differentially expressed in the ANOVA analysis.

	D1	D2	D3	D4
**D1**	-	0.004	0.004	0.19
**D2**	3.21	-	0.99	0.01
**D3**	3.21	0.24	-	0.01
**D4**	1.45	3.03	2.93	-

D1 = standard diet for growing-finishing pigs; D2 = standard diet supplemented with linseed (source of *n-*3 PUFA); D3 = standard diet supplemented with linseed, vitamin E and selenium (250 and 0.31 mg *per* kg of feed respectively; D4 = standard diet supplemented with linseed and plant extracts from grape-skin (3 g *per* kg of feed) and oregano (2 g *per* kg of feed) as source of polyphenols (total polyphenols added 37.6 mg *per* kg of feed).

**Fig 2:**
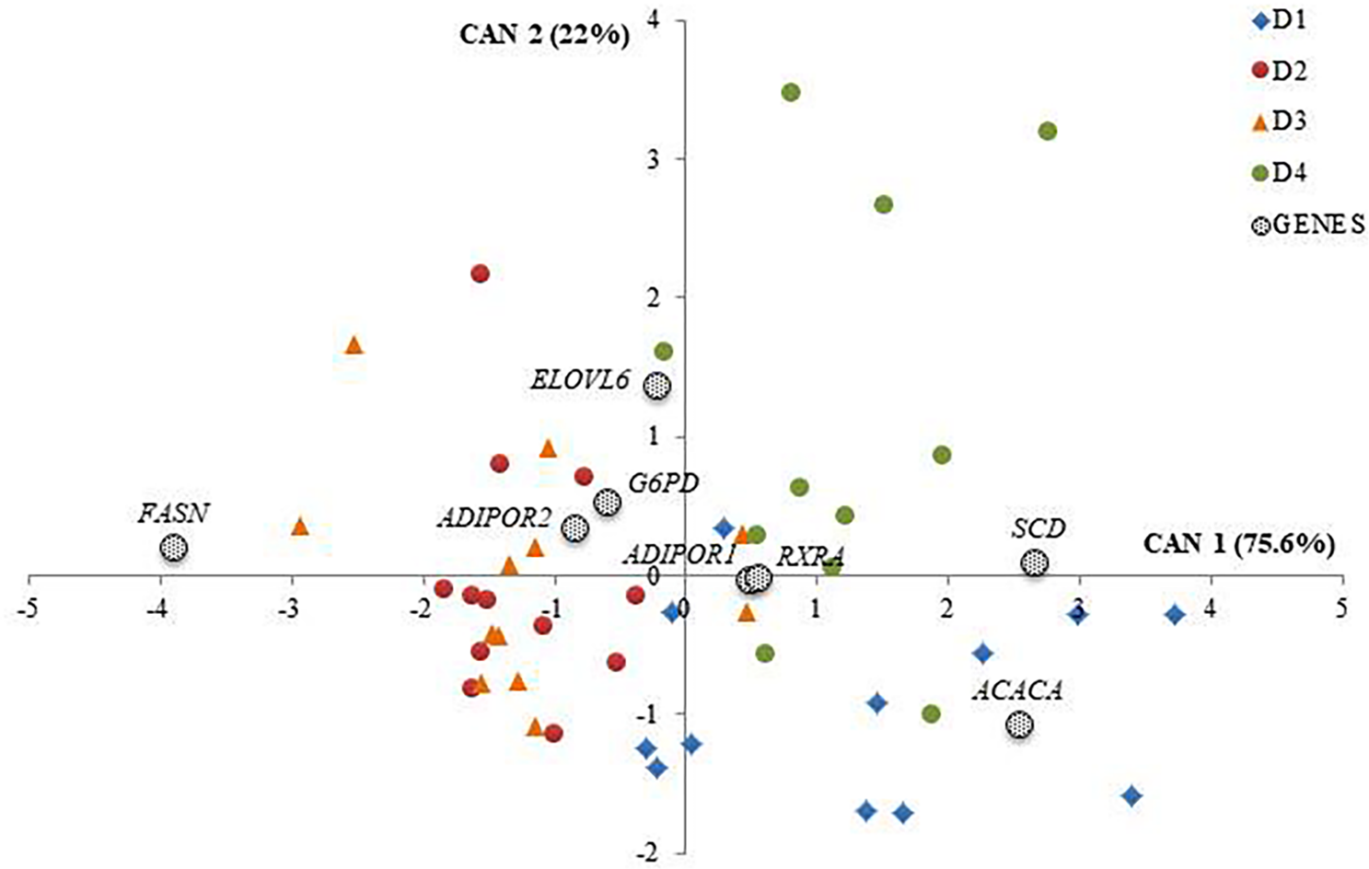
Canonical discriminant analysis (CDA) performed on stepwise-selected genes. D1 = standard diet for growing-finishing pigs; D2 = standard diet supplemented with linseed (source of *n-*3 PUFA); D3 = standard diet supplemented with linseed, vitamin E and selenium; D4 = standard diet supplemented with linseed and plant extracts from grape-skin and oregano as a source of polyphenols.

Then, SDA retained 8 out of the 12 DEGs namely *FASN*, *ELOVL6*, *SCD*, *ACACA*, *ADIPOR1*, *ADIPOR2*, *G6PD* and *RXRA*. These genes were used to perform a second CDA analysis. Results of Mahalanobis’s distances and Hotelling’s T-test confirmed that the effect on gene expression did not differ between D2 and D3 (*P* = 0.9950), whereas it was different between D4 and D1 (*P* = 0.0035). Moreover, the gene expression differs when comparing D4 to D2 and to D3 (*P* = 0.0006 and *P* = 0.0001 respectively) and when D1 was compared to D2 and to D3 (P = 0.0019 and P < 0.0001 respectively) (Table 4). The two CANs, were able to explain the 97.6% of the total variation (where CAN1 explained the 75.6% of the total variation and CAN2 explained the 22%), revealing significant differences among diets for the expression of the considered genes, as displayed in Fig 2. In Fig 2, the scores of each animal were plotted in the space of the first two CANs. In the same figure, the canonical coefficients of the 8 selected genes (dotted circles) were also reported. In particular, *FASN*, *SCD*, *ACACA* and *ELOVL6* had, in absolute value, canonical coefficients greater than 1 and, in consequence, their expression was strongly influenced by the diet supplied (Fig 2). Indeed, considering CAN1, *FASN*, *SCD* and *ACACA* expressions had the greatest discriminating power among diets and allowed to distinguish D2 and D3 from D1 and D4, respectively. Moreover, in CAN2, *ELOVL6* and *ACACA* were the most discriminants between D4 and D1. No separation was found between D2 and D3 in CAN2.

**Table 4:** Hotelling’s T-test *P*-values (above diagonal) and Mahalanobis’s distances (below diagonal) between diets, calculated on the expression of a set of 8 genes selected by the stepwise discriminant analysis.

	D1	D2	D3	D4
**D1**	-	0.002	< .0001	0.003
**D2**	3.84	-	0.13	0.001
**D3**	7.30	1.68	-	0.0001
**D4**	3.50	4.51	5.42	-

D1 = standard diet for growing-finishing pigs; D2 = standard diet supplemented with linseed (source of *n-*3 PUFA); D3 = standard diet supplemented with linseed, vitamin E and selenium; D4 = standard diet supplemented with linseed and plant extracts from grape-skin and oregano as a source of polyphenols.

## Discussion

In the present study, both univariate and multivariate analysis was applied in order to investigate the influence of four different diets on the expression level of a set of genes in porcine muscle. Genes were selected because of their role in lipid and energy metabolism and in the fat deposition of muscle tissue. Besides univariate statistics, which considers the effect of a single gene expression, multivariate analysis was conducted to account for the relationship (covariance and correlation) among the expressed genes, making the two approaches, complementary. Indeed, since the transcription levels of genes involved in the same metabolic pathways are often highly correlated, there is a growing trend to analyze gene expression data utilizing both univariate and multivariate approaches [27, 28].

Results from the multivariate analysis displayed overall the presence of two patterns of genes differently placed in Fig 2. The first included genes involved in FA synthesis [29], namely *FASN*, *ACACA*, *SCD*, *ELOVL6*, showing the highest difference among diets (canonical coefficients > 1). The second one, having canonical coefficients < 1, was more related to β-oxidation processes and involves the expression of *ADIPOR1*, *ADIPOR2*, *RXRA* and *G6PD*, which is involved in NADH synthesis from glucose [1].

Moreover, multivariate analysis evidenced that, considering the CAN1, gene expression values of pigs fed diets D2 and D3 clustered together, thus the two diets produced a similar effect on the expression of the target genes; while D4 and D1 are separated (and so different) from D2 and D3. The genes whose expression mostly differs between D4-D1 and D2-D3 were *SCD*, *ACACA*, *FASN*, *RXRA*, *ADIPOR1* and *ADIPOR2*. Thus, multivariate analysis separated D4-D1 from D2-D3 on the CAN1, and genes mostly influencing this difference were those involved in lipogenesis (canonical coefficient > 1), unless genes responsible for β-oxidation were also involved to a lesser extent.

With regards to D2-D3 comparison, both multivariate and ANOVA analysis did not show differences between the two diets, assuming that vitamin E and selenium added to the diet (D3) did not produce any different effect on the expression of the tested genes if compared to a diet rich in *n-*3 PUFA (D2). Indeed, the expression level of *FASN*, *ACACA* and *SCD* were lower in D2 and D3 compared to D1 and D4 making to suppose a reduced *de novo* lipogenesis in D2 and D3 likely related to the dietary PUFA addition.

Considering the differences observed between D1 and D2-D3 by the multivariate analysis, we can suppose that the lower expression of *FASN*, *ACACA* and *SCD* can be due to the dietary supplementation of essential FA that will be directly stored in the tissue, instead of activating *de novo* FA synthesis. In fact, the inhibitory effect of PUFA on *de novo* lipogenesis is actually well known in mice and human liver so far [30] unless, to the best of Authors’ knowledge, no similar studies were reported in swine muscle. The reduction of the expression level of lipogenic genes, administering a diet supplemented with *n-*3 PUFA, was recently reported in a study [31] comparing the transcription level of high-fat *vs* low-fat diet in poultry liver. According to the study of Desert et al. [31] the addition of *n-*3 PUFA to the diet did not activate, compared to a standard diet, *de novo* lipogenic processes thus the lower expression of *FASN*, *ACACA* and *SCD* in a diet high in *n-*3 PUFA is due to the dietary supplementation of essential FA. Moreover, in accord with our study, the same Authors [31] observed a down-regulation of genes involved in β-oxidation, even if the study was developed in the liver of chicken fed high-fat diet compared to a standard diet. Nevertheless, results by ANOVA reported that *ADIPOR1* was the only DE gene in D1 compared to D2. The increased expression of *ADIPOR1* in D1 was found to stimulate the expression of genes involved in *de novo* lipogenesis by increasing glucose uptake in human myocytes [32]. Moreover, in support of our result, overall, dietary *n-*3 PUFA were reported to reduce adiponectin plasma content in humans so far [33,34], while opposite results were sometimes reported in the literature, mainly due to deep differences in the experimental conditions among studies [33].

About D2-D4 comparison, multivariate analysis displayed that the two diets influenced gene expressions in different ways (Fig 2). In accord with this, ANOVA analysis showed the largest number of DEGs in D4-D2 comparison (Fig 1), with all the DEGs displaying a higher expression in D4. The DEGs that in D4 showed an increased expression can be functionally clustered as follows: two key regulatory genes of lipid and glucose metabolism (*CHREBP* and *PPARA*) [35,36]; lipogenic genes (*FASN*, *ACACA*, *ELOVL6* and *SCD*); genes involved in β-oxidation (*ADIPOQ*, *ADIPOR1*, *ADIPOR2*, *RXRA*) and *PLIN2*, involved in the storage and utilization of intracellular lipid droplets in skeletal muscle [37–39]. *ACACA*, *FASN*, *ELOVL6* and *SCD* are indicated as lipogenic genes since they code for enzymes playing a key role in *de novo* FA synthesis, and their expression is regulated primarily by *SREBP1C* (*SREBF1*) and *CHREBP* genes [40]. In the present study, *SREBP1C* was not differentially expressed among diets, while the expression of *CHREBP* was found increased in D4 compared to D2. This result may suggest the activation of the *CHREBP* signalling pathway, which is reported to stimulate the expression of *ACACA* and *FASN* genes (both coding for enzymes catalyzing steps of FA synthesis) [35]. This pathway can also promote the elongation and desaturation of FA carbon chain through the activation of *ELOVL6* and *SCD* in porcine adipocyte cell cultures added with glucose [41] and in mice liver [40,42]. The *CHREBP* signalling pathway is known to increase dietary glucose uptake and to stimulate genes involved in lipogenesis also independently from insulin stimulation [35,43]. This feature may be crucial to understanding why, in the present study, D4 activated *CHREBP* expression and did not stimulate the expression of *SREBP1C*, a gene whose expression is insulin-dependent [43]. In literature diets supplemented with polyphenols and PUFA were found in human to suppress insulin-mediated lipogenic pathways [44]. Moreover, resveratrol was reported to be associated to glucose uptake in skeletal muscle [45] thus suggesting that, in the present study, plant-derived polyphenols may suppress insulin-dependent FA synthesis, but not insulin-independent lipogenesis through the activation of *CHREBP* signalling pathway. In D4, also genes involved in β-oxidation were more expressed compared to D2. The increased expressions of *ADIPOQ* and its binding receptors *ADIPOR1* and *ADIPOR2* are known to stimulate *PPARA* expression in human and mouse skeletal muscle and liver [36,46,47] and to activate the PPARA-RXRA complex, which increases lipid catabolism and FA oxidation. In agreement with our results, Park et al. [48] reported that resveratrol, a polyphenolic compound in grape-skin, increased *ADIPOR1* and *ADIPOR2* expression in mice renal cortex, and recent studies described an increased expression of *PPARA* induced by the diet supplementation of both long-chain PUFA and polyphenols in human and mice liver [49,50]. Indeed, the D4 seemed to stimulate the expression of genes associated with lipogenic and β-oxidation processes, regulated by *CHREBP* and *PPARA*, respectively. The co-activation of lipogenesis and β-oxidation in the same tissue was previously observed in knockout mice liver by Chakravarthy et al. [51], who reported that products derived by *de novo* FA synthesis can activate *PPARA* with the aim to maintain glucose, lipid and cholesterol homeostasis. In addition, a research by Wang et al. [52] in mice liver evidenced that both *PPARA* and *CHREBP* play a role on *ELOVL6* and *SCD* expression control, contributing to *de novo* lipogenesis of monounsaturated FA addressed to maintain the cellular neutral lipid fraction [52]. Also a study from Revilla et al. [53] observed that in porcine adipose tissue not only PPARs can regulate lipogenic genes expression, but also a *trans-* eQTL identified for *ELOVL6* resulted associated with *SCD* expression, supporting an involvement of common regulatory elements. Lastly, in the present study, *PLIN2* was significantly more expressed in D4 compared to D2. In literature both FA synthesis and β-oxidation were found to stimulate *PLIN2*, a gene of the Perilipins family involved in the storage and utilization of intracellular lipid droplets in skeletal muscle metabolism [37–39]. *PLIN2* is also an adipocyte precursor [54] and can influence glucose metabolism [39]. In accord with our results, it has been reviewed that both *ELOVL6* and *SCD* genes involved in long-chain FA synthesis (especially *ELOVL6* for oleic acid) [31], and *PPARA* can stimulate the expression of *PLIN2* [54].

Multivariate analysis allowed also to observe differences between D4 and D1 considering the CAN2, where genes mostly contributing to this difference were *ELOVL6* and *ACACA* (canonical coefficient > 1) related to monounsaturated FA synthesis, and *G6PD* (Fig 2). Indeed, ANOVA analysis showed that *ELOVL6* and *G6PD* were more expressed in presence of *n-*3 PUFA and polyphenols supplementations (D4) compared to the standard diet (D1). As described above, *ELOVL6* plays a role in FA chain elongation, and thus its increased expression in D4 leads to suppose that the combination of plant extract (source of polyphenols) and linseed (source of *n-*3 PUFA) may stimulate long chain FA synthesis. Furthermore, Kamei et al. [55] found an increased expression of both *ELOVL6* and *SCD* in the liver of mice fed high-fat diet supplemented with polyphenols. This result led the Authors to suppose that the higher expression of *ELOVL6* and *SCD* might result in an increased synthesis of PUFA and in a reduced level of saturated FAs. Since we have found similar results in D4 compared to the other diets, it is, therefore, possible to hypothesize a more active pattern of FAs desaturation in the muscle tissue of pigs fed D4. Moreover, the higher expression of *G6PD* may contribute both to stimulate lipogenesis [1] and to protect the cells from the oxidative stress [56]. Interestingly, an increased *G6PD* expression was observed also in D3 compared to D1. This evidence suggests an important role played by both vitamin E and polyphenols in increasing *G6PD* expression to maintain cellular redox balance, as reported by Ho et al. [56] in human fibroblast.

To summarize, the results demonstrate that the addition of *n-*3 PUFA alone or *n-*3 PUFA and plant extract leads to deep differences in the expression of the tested genes. On the contrary, adding *n-*3 PUFA with vitamin E and selenium did not produce a different effect compared to the unique addition of *n-*3 PUFA. The difference in the effect should be imputable to the different components of the antioxidant integration. Indeed it has been reviewed that a mixture of polyphenolic compounds may accomplish the greater phenotypic effect [57]. Nevertheless other Authors reported contrasting results, because the knowledge on the *in vivo* biological polyphenols effects are poorly known in livestock animals at present time [18, 58]. The present study evidenced an increased *de novo* FA synthesis in the diet supplemented with plant extract (source of polyphenols) and linseed (source of *n-*3 PUFA) (D4), while the addition of *n-*3 PUFA alone did not stimulate genes involved in FA synthesis. Moreover, D4 appeared to stimulate *de novo* lipogenesis of long-chain FA synthesis, β-oxidation processes, FA myocyte lipid storage and to promote cell protection from oxidative stress. The cellular protection from the oxidative stress may be promoted also by the diet enriched with *n-*3 PUFA, vitamin E and selenium, compared to the standard diet, while the expression of genes related to FA synthesis seems not to be influenced by this diet.

The increased expression of lipogenic genes in D4 compared to the other diets could be explained with the role played by both polyphenols and *n-*3 PUFA in stimulating glucose uptake in *Longissimus thoracis* muscle, which can activate *CHREBP* thus stimulating lipogenic genes for *de novo* FA synthesis. Moreover, the expression of genes involved in β-oxidation might be considered useful in maintaining muscle homeostasis. In the light of these hypotheses, it will be of interest to further validate the results on a larger group of animals and to account also the muscle FA composition in pigs fed different diets.

## Conclusion

The results obtained in the present study are useful to increase knowledge about the effects determined by experimental diets enriched in PUFA and antioxidants on the expression of skeletal muscle genes in pigs. Overall the dietary treatments produced effects on the expression of the tested genes. The use of both multivariate CDA and univariate ANOVA analyses allowed to better identify the gene interactions related to biological processes. Indeed, the results suggested that adding plant extract (source of polyphenols) and linseed (source of *n-*3 PUFA) stimulates the expression of genes involved in the control of muscle metabolism, leading to a mutual interaction between lipogenesis and oxidative processes in the *Longissimus thoracis* muscle of pigs. This stimulation was more evident in the diet supplemented with polyphenols and *n-*3 PUFA compared to a diet only enriched in *n-*3 PUFA. On the contrary, the integration of vitamin E and Selenium in the diet did not significantly alter the expression of the tested genes in comparison to the diet with only linseed supplementation. These results were a first step in the formulation of functional diets for pigs addressing consumers’ demand for healthy meat products.

## Supporting information

S1 Fig Canonical discriminant analysis (CDA) performed on differentially expressed genes (DEGs) by ANOVA analysis. **Samples were distributed according to the canonical coefficients in the two axes (CAN 1 and CAN2).** Legend: D1 = standard diet for growing-finishing pigs; D2 = standard diet supplemented with linseed (source of n-3 PUFA); D3 = standard diet supplemented with linseed, vitamin E and selenium; D4 = standard diet supplemented with linseed and plant extracts from grape-skin and oregano as source of polyphenols.
(TIF)

S1 Table Summary of the name, functions and references of each candidate gene in the study. (DOC)

S2 Table List of the genes used in this study. TM = Annealing temperature.
(DOC)

S3 Table Expression levels of the target genes. *P*-values of the comparisons are reported on the top of each cell and the means and standard errors for each diet are shown between brackets. Legend: D1 = standard diet for growing-finishing pigs; D2 = standard diet supplemented with linseed; D3 = standard diet supplemented with linseed, vitamin E and selenium; D4 = standard diet supplemented with linseed and plant extracts from grape-skin and oregano as source of polyphenols.
(DOC)

S1 File Complete datasets used for the statistical analyses included in Tables A-C. Table A: Single expression data (generally three quantification per gene) of all the target and houskeeping genes, with means, standard deviations and coefficient of variation of the three quantifications. Table B: The expression data (mean of three quantifications) normalized using the geometric mean of the selected reference genes. Table C: The raw expression data calculated as mean of three quantifications.
(XLSX)
