# Supplementary figures and images for: Effect of dietary polyunsaturated fatty acid and antioxidant supplementation on the transcriptional level of genes involved in lipid and energy metabolism in swine
Source: PLoS One. 2018 Oct 4;13(10):e0204869. doi: 10.1371/journal.pone.0204869 (PMC6171869; doi:10.1371/journal.pone.0204869)

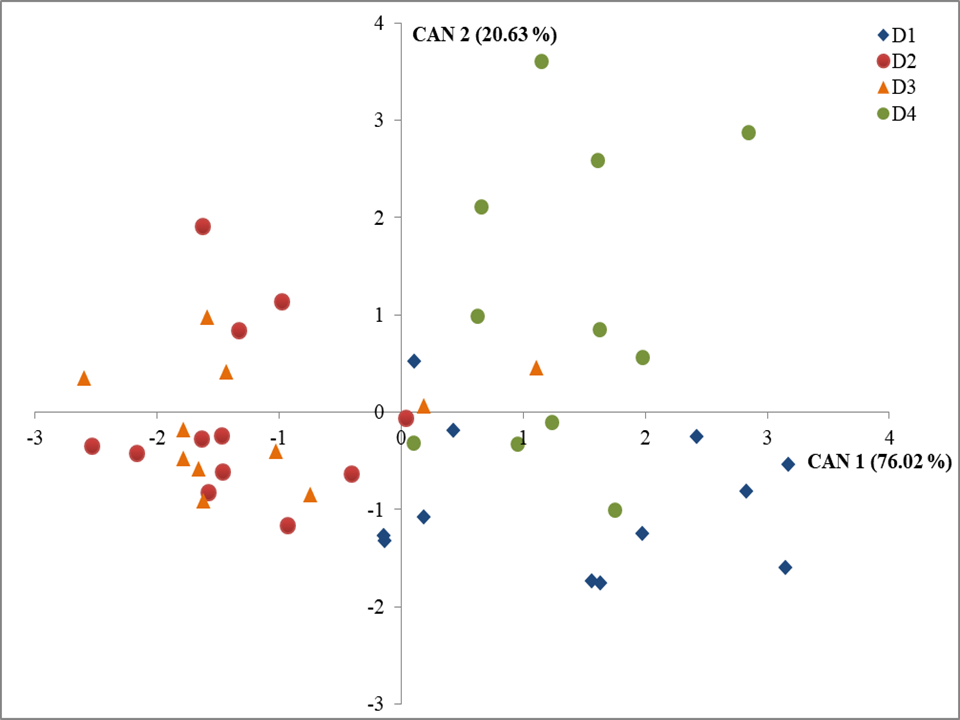

Supplement: S1 Fig — Samples were distributed according to the canonical coefficients in the two axes (CAN 1 and CAN2). Legend: D1 = standard diet for growing-finishing pigs; D2 = standard diet supplemented with linseed (source of n-3 PUFA); D3 = standard diet supplemented with linseed, vitamin E and selenium; D4 = standard diet supplemented with linseed and plant extracts from grape-skin and oregano as source of polyphenols. (TIF) [file pone.0204869.s001.tif]
