# Supplementary material for: Effect of dietary polyunsaturated fatty acid and antioxidant supplementation on the transcriptional level of genes involved in lipid and energy metabolism in swine
Source: PLoS One. 2018 Oct 4;13(10):e0204869. doi: 10.1371/journal.pone.0204869 (PMC6171869; doi:10.1371/journal.pone.0204869)
Supplement: S1 Table — (DOC) [file pone.0204869.s002.doc]

| **Gene** | **full name** | **Functions** | **References** |
| --- | --- | --- | --- |
| ***ACACA*** | Acetyl-Coenzyme A Carboxylase Alpha | Metabolism of water-soluble vitamins and cofactors and fatty acid biosynthesis initiation. GO annotations related to this gene include ligase activity and D-alanine-D-alanine ligase activity. | [1-7] |
| ***ADIPOQ*** | Adiponectin, C1Q and Collagen Domain-Containing | Important adipokine involved in the control of fat metabolism and insulin sensitivity, with direct anti-diabetic, anti-atherogenic and anti-inflammatory activities. GO annotations related to this gene include *protein homodimerization activity* and *receptor binding*. | [1,8-10] |
| ***ACLY*** | ATP Citrate Lyase | The primary enzyme responsible for the synthesis of cytosolic acetyl-CoA in various tissues. It has a central role in de novo lipid synthesis. GO annotations related to this gene include cofactor binding and ATP citrate synthase activity. | [3] |
| ***ADIPOR1*** | Adiponectin Receptor protein1 | Receptor for ADIPOQ, an essential hormone secreted by adipocytes, regulates glucose and lipid metabolism. GO annotations related to this gene include identical protein binding and protein kinase binding. | [1,8,11-14] |
| ***ADIPOR2*** | Progestin And AdipoQ Receptor Family Member II | Receptor for ADIPOQ, an essential hormone secreted by adipocytes, regulates glucose and lipid metabolism. Required for normal body fat and glucose homeostasis.GO annotations related to this gene include identical protein binding and receptor activity. | [1,6-11] |
| ***ATGL*** | Adipose Triglyceride Lipase | Catalyzes the initial step in triglyceride hydrolysis in adipocyte and non-adipocyte lipid droplets. Also it has acylglycerol transacylase activity. GO annotations related to this gene include triglyceride lipase activity. | [15] |
| ***CHREBP*** | Carbohydrate-responsive element-binding protein; | Carbohydrate Metabolism and Insulin Resistance. GO annotations related to this gene include transcription factor activity, sequence-specific DNA binding and protein heterodimerization activity. | [3,16-20] |
| ***ELOVL6*** | Elongation Of Long Chain Fatty Acids, Member 6, | Catalyzes the first and rate-limiting reaction of the four that constitute the long-chain fatty acids elongation cycle. This endoplasmic reticulum-bound enzymatic process allows the addition of 2 carbons to the chain of long- and very long-chain fatty acids/VLCFAs per cycle. GO annotations related to this gene include transferase activity and transferase activity, transferring acyl groups other than amino-acyl groups. | [1,3,18,19,21,22] |
| ***FADS2*** | Fatty Acid Desaturase 2; | Component of a lipid metabolic pathway that catalyses the biosynthesis of highly unsaturated fatty acids from precursor essential polyunsaturated fatty acids linoleic acid and alpha-linolenic acid. GO annotations related to this gene include iron ion binding and oxidoreductase activity, acting on paired donors, with oxidation of a pair of donors resulting in the reduction of molecular oxygen to two molecules of water. | [3,21-23] |
| ***FASN*** | Fatty Acid Synthase | Catalyzes the formation of long-chain fatty acids from acetyl-CoA, malonyl-CoA and NADPH. GO annotations related to this gene include poly(A) RNA binding and identical protein binding. | [1,2,4,6,15,22,24-27] |
| ***G6PD*** | Glucose-6-Phosphate Dehydrogenase | Catalyzes the rate-limiting step of the oxidative pentose-phosphate pathway, which represents a route for the dissimilation of carbohydrates besides glycolysis. The main function of this enzyme is to provide reducing power (NADPH) and pentose phosphates for fatty acid and nucleic acid synthesis. GO annotations related to this gene include protein homodimerization activity and carbohydrate binding. | [3,6,25] |
| ***LIPE*** | Lipase E, Hormone Sensitive Type | In adipose tissue and heart, it primarily hydrolyzes stored triglycerides to free fatty acids, while in steroidogenic tissues, it principally converts cholesteryl esters to free cholesterol for steroid hormone production. GO annotations related to this gene include protein kinase binding and triglyceride lipase activity. | [15,28] |
| ***LPL*** | Lipoprotein Lipase | The primary function of this lipase is the hydrolysis of triglycerides of circulating chylomicrons and very low-density lipoproteins (VLDL). GO annotations related to this gene include receptor binding and carboxylic ester hydrolase activity. | [2,15,25] |
| ***LXRA*** | Liver X Receptor-Alpha; | Nuclear receptor. Interaction with RXR shifts RXR from its role as a silent DNA-binding partner to an active ligand-binding subunit in mediating retinoid responses through target genes defined by LXRES. GO annotations related to this gene include transcription factor activity, sequence-specific DNA binding and transcription coactivator activity. | [18-20,29] |
| ***ME1*** | Malic Enzyme 1 | Metabolism and Regulation of lipid metabolism by Peroxisome proliferator-activated receptor alpha (PPARalpha). GO annotations related to this gene include *electron carrier activity* and *NAD binding*. | [3,5,6,25] |
| ***MGLL*** | Monoglyceride Lipase | Converts monoacylglycerides to free fatty acids and glycerol. GO annotations related to this gene include protein homodimerization activity and lipid binding. | [15] |
| ***PLIN2*** | Perilipin 2 | Metabolism and Regulation of lipid metabolism by Peroxisome proliferator-activated receptor alpha (PPARalpha). Involved in development and maintenance of adipose tissue. | [30,31] |
| ***PLIN3*** | Perilipin 3 | Required for the transport of mannose 6-phosphate receptors (MPR) from endosomes to the trans-Golgi network. | [31] |
| ***PLIN5*** | Perilipin 5 | Lipid droplet-associated protein that maintains the balance between lipogenesis and lipolysis and also regulates fatty acid oxidation in oxidative tissues. Recruits mitochondria to the surface of lipid droplets and is involved in lipid droplet homeostasis by regulating both the storage of fatty acids in the form of triglycerides and the release of fatty acids for mitochondrial fatty acid oxidation. GO annotations related to this gene include identical protein binding and lipase binding. | [32] |
| ***PPARA*** | Peroxisome Proliferator Activated Receptor Alpha | Ligand-activated transcription factor. Key regulator of lipid metabolism. Activated by the endogenous ligand 1-palmitoyl-2-oleoyl-sn-glycerol-3-phosphocholine. GO annotations related to this gene include transcription factor activity, sequence-specific DNA binding and receptor activity. | [2,3,8,10,13,15,17,18,26,33-37] |
| ***PPP3CA*** | Protein Phosphatase 3 Catalytic Subunit Alpha | Calcium-dependent, calmodulin-stimulated protein phosphatase. Many of the substrates contain a PxIxIT motif. This subunit may have a role in the calmodulin activation of calcineurin. GO annotations related to this gene include calcium ion binding and enzyme binding. | [28] |
| ***RXRA*** | Retinoid X Receptor Alpha | Receptor for retinoic acid. Regulate gene expression in various biological processes. The RXRA/PPARA heterodimer is required for PPARA transcriptional activity on fatty acid oxidation genes such as ACOX1 and the P450 system genes. GO annotations related to this gene include transcription factor activity, sequence-specific DNA binding and protein heterodimerization activity. | [3,17,25] |
| ***SCD*** | Stearoyl-CoA Desaturase | Stearyl-CoA desaturase that utilizes O(2) and electrons from reduced cytochrome b5 to introduce the first double bond into saturated fatty acyl-CoA substrates. GO annotations related to this gene include iron ion binding and stearoyl-CoA 9-desaturase activity. | [1-6, 18,24,26,38] |
| ***SREBP1C*** | Sterol Regulatory Element Binding Transcription Factor 1 | Transcriptional activator required for lipid homeostasis. Regulates transcription of the LDL receptor gene as well as the fatty acid and to a lesser degree the cholesterol synthesis pathway. GO annotations related to this gene include transcription factor activity, sequence-specific DNA binding and chromatin binding. | [2,15-18,20,25,33,34,39] |

**Literature cited in supplementary material:**

1. Cánovas A, Quintanilla R, Amills M, Pena RN. Muscle transcriptomic profiles in pigs with divergent phenotypes for fatness traits. BMC Genomics. 2010;11: 372.

2. Tous N, Theil PK, Lauridsen C, Lizardo R, Vilà B, Esteve-Garcia E. Dietary conjugated linoleic acid modify gene expression in liver, muscles, and fat tissues of finishing pigs. J Anim Sci. 2012;90: 340–342.

3. Corominas J. Functional genomics and candidate genes for meat quality traits in pigs. Ph.D Thesis, Universitat Autònoma de Barcelona. 2013. Available from: https://www.tesisenred.net/handle/10803/129080

4. Madeira MS, Pires VMR, Alfaia CM, Costa ASH, Luxton R, Doran O, et al. Differential effects of reduced protein diets on fatty acid composition and gene expression in muscle and subcutaneous adipose tissue of Alentejana purebred and Large White × Landrace × Pietrain crossbred pigs. Br J Nutr. 2013;110: 216–229.

5. Benítez R, Núñez Y, Fernández A, Isabel B, Rodríguez C, Daza A, et al. Adipose tissue transcriptional response of lipid metabolism genes in growing Iberian pigs fed oleic acid v. carbohydrate enriched diets. Animal. 2016;10: 939–946.

6. Guillevic M, Kouba M, Mourot J. Effect of a linseed diet or a sunflower diet on performances, fatty acid composition, lipogenic enzyme activities and stearoyl-CoA-desaturase activity in the pig. Livest Sci. 2009;124: 288–294.

7. Zhang W, Zhang J, Cui L, Ma J, Chen C, Ai H, et al. Genetic architecture of fatty acid composition in the longissimus dorsi muscle revealed by genome-wide association studies on diverse pig populations. Genet Sel Evol. 2016 ; 48 : 5.

8. Pawlak M, Lefebvre P, Staels B. Molecular mechanism of PPARα action and its impact on lipid metabolism, inflammation and fibrosis in non-alcoholic fatty liver disease. J Hepatol. 2015;62: 720–733.

9. Barnea M, Shamay A, Stark AH, Madar Z. A high-fat diet has a tissue-specific effect on adiponectin and related enzyme expression. Obesity. 2006;14: 2145–2153.

10. Marette A, Liu Y, Sweeney G. Skeletal muscle glucose metabolism and inflammation in the development of the metabolic syndrome. Rev Endocr Metab Disord. 2014;15: 299–305.

11. Barnea M, Shamay A, Stark AH, Madar Z. A High-Fat Diet Has a Tissue-Specific Effect on Adiponectin and Related Enzyme Expression. Obesity. 2006;14: 2145-2153.

12. de Oliveira C, de Mattos AB, Biz C, Oyama LM, Ribeiro EB, Oller do Nascimento C. High-fat diet and glucocorticoid treatment cause hyperglycemia associated with adiponectin receptor alterations. Lipids Health Dis. 2011;10: 11.

13. Caselli C. Role of adiponectin system in insulin resistance. Mol Genet Metab. 2014;113: 155–60.

14. Mazaherioun M, Saedisomeolia A, Javanbakht MH, Koohdani F, Eshraghian MR, Djalali M. Beneficial effects of n-3 polyunsaturated fatty acids on adiponectin levels and AdipoR gene expression in patients with type 2 diabetes mellitus: A randomized, placebo-controlled, double-blind clinical trial. Arch Med Sci . 2017;13: 716–724.

15. Huang CW, Chen YJ, Yang JT, Chen CY, Ajuwon KM, Chen SE, et al. Docosahexaenoic acid increases accumulation of adipocyte triacylglycerol through up-regulation of lipogenic gene expression in pigs. Lipids Health Dis. 2017;16:33.

16. Dentin R, Benhamed F, Hainault I, Fauveau V, Foufelle F, Dyck JRB, Girard J, Postic C. Liver-specific inhibition of ChREBP improves hepatic steatosis and insulin resistance in ob/ob mice. Diabetes. 2006;55: 2159–2170.

17. Gondret F, Vincent A, Houée-Bigot M, Siegel A, Lagarrigue S, Louveau I, et al. Molecular alterations induced by a high-fat high-fiber diet in porcine adipose tissues: variations according to the anatomical fat location. BMC Genomics. BMC Genomics. 2016;17: 120.

18. Wang Y, Botolin D, Xu J, Christian B, Mitchell E, Jayaprakasam B, et al. Regulation of hepatic fatty acid elongase and desaturase expression in diabetes and obesity. J Lipid Res. 2006;47: 2028–2041.

19. Sun H, Jiang T, Wang S, He B, Zhang Y, Piao D, et al. The effect of LXRα, ChREBP and Elovl6 in liver and white adipose tissue on medium- and long-chain fatty acid diet-induced insulin resistance. Diabetes Res Clin Pract. 2013;102: 183–192.

20. Zhang C, Luo J, Yu B, Zheng P, Huang Z, Mao X, et al. Dietary resveratrol supplementation improves meat quality of finishing pigs through changing muscle fiber characteristics and antioxidative status. Meat Sci. 2015;102: 15–21.

21. González-Calvo L, Joy M, Blanco M, Dervishi E, Molino F, Sarto P, et al. Effect of vitamin E supplementation or alfalfa grazing on fatty acid composition and expression of genes related to lipid metabolism in lambs. J Anim Sci. 2015;93: 3044–3054.

22. Szostak A, Ogłuszka M, Te Pas MFW, Poławska E, Urbański P, Juszczuk-Kubiak E, et al. Effect of a diet enriched with omega-6 and omega-3 fatty acids on the pig liver transcriptome. Genes Nutr. 2016;11: 9.

23. Renaville B, Prandi A, Fan B, Sepulcri A, Rothschild MF, Piasentier E. Candidate gene marker associations with fatty acid profiles in heavy pigs. Meat Sci. 2013;93: 495–500.

24. Madeira MS, Pires VMR, Alfaia CM, Luxton R, Doran O, Bessa RJB, et al. Combined effects of dietary arginine, leucine and protein levels on fatty acid composition and gene expression in the muscle and subcutaneous adipose tissue of crossbred pigs. Br J Nutr. 2014;111: 1521–1135.

25. De Tonnac A, Labussière E, Vincent A, Mourot J. Effect of α-linolenic acid and DHA intake on lipogenesis and gene expression involved in fatty acid metabolism in growing-finishing pigs. Br J Nutr. 2016;116: 7–18.

26. Inoue N, Nagao K, Wang Y-M, Noguchi H, Shirouchi B, Yanagita T. Dietary conjugated linoleic acid lowered tumor necrosis factor-alpha content and altered expression of genes related to lipid metabolism and insulin sensitivity in the skeletal muscle of Zucker rats. J Agric Food Chem . 2006 ;54: 7935–7939.

27. Zhang Y, Li D, Sun B. Do housekeeping genes exist? PLoS One. 2015;10: e0123691.

28. Park HS, Lim JH, Kim MY, Kim Y, Hong YA, Choi SR, et al. Resveratrol increases AdipoR1 and AdipoR2 expression in type 2 diabetic nephropathy. J Transl Med. 2016 ;14: 176.

29. Corominas J, Ramayo-Caldas Y, Puig-Oliveras A, Estellé J, Castelló A, Alves E, et al. Analysis of porcine adipose tissue transcriptome reveals differences in de novo fatty acid synthesis in pigs with divergent muscle fatty acid composition. BMC Genomics. 2013;14: 843.

30. Davoli R, Gandolfi G, Braglia S, Comella M, Zambonelli P, Buttazzoni L, et al. New SNP of the porcine Perilipin 2 (PLIN2) gene, association with carcass traits and expression analysis in skeletal muscle. Mol Biol Rep. 2011;38: 1575–1583.

31. Lecchi C, Invernizzi G, Agazzi A, Modina S, Sartorelli P, Savoini G, et al. Effects of EPA and DHA on lipid droplet accumulation and mRNA abundance of PAT proteins in caprine monocytes. Res Vet Sci . 2013;94: 246–251.

32. Zappaterra M, Mazzoni M, Zambonelli P, Davoli R. Investigation of the Perilipin 5 gene expression and association study of its sequence polymorphism with meat and carcass quality traits in different pig breeds. Animal. 2018;12: 1135-1143.

33. de Souza CO, Teixeira AAS, Biondo LA, Lima Junior EA, Batatinha HAP, Rosa Neto JC. Palmitoleic Acid Improves Metabolic Functions in Fatty Liver by PPARα-Dependent AMPK Activation. J Cell Physiol. 2017;232: 2168–2177.

34. Chakravarthy MV, Pan Z, Zhu Y, Tordjman K, Schneider JG, Coleman T, et al. “New” hepatic fat activates PPARα to maintain glucose, lipid, and cholesterol homeostasis. Cell Metab. 2005;1: 309–322.

35. Domínguez-Avila J, González-Aguilar G, Alvarez-Parrilla E, de la Rosa L. Modulation of PPAR Expression and Activity in Response to Polyphenolic Compounds in High Fat Diets. Int J Mol Sci. 2016;17: E1002.

36. Pawar A, Botolin D, Mangelsdorf DJ, Jump DB. The Role of Liver X Receptor-α in the Fatty Acid Regulation of Hepatic Gene Expression. J Biol Chem. 2003;278: 40736-40743.

37. Di Nunzio M, Danesi F, Bordoni A. N-3 PUFA as regulators of cardiac gene transcription: A new link between ppar activation and fatty acid composition. Lipids. 2009;44: 1073-1079.

38. Pena RN, Ros-Freixedes R, Tor M, Estany J. Genetic marker discovery in complex traits: A field example on fat content and composition in pigs. Int J Mol Sci. 2016;14: E2100.

39. Haas JT, Miao J, Chanda D, Wang Y, Zhao E, Haas ME, et al. Hepatic insulin signaling is required for obesity-dependent expression of SREBP-1c mRNA but not for feeding-dependent expression. Cell Metab. 2012;15: 873–884.
