# Supplementary material for: Effect of dietary polyunsaturated fatty acid and antioxidant supplementation on the transcriptional level of genes involved in lipid and energy metabolism in swine
Source: PLoS One. 2018 Oct 4;13(10):e0204869. doi: 10.1371/journal.pone.0204869 (PMC6171869; doi:10.1371/journal.pone.0204869)
Supplement: S2 Table — TM = Annealing temperature. (DOC) [file pone.0204869.s003.doc]

| **Gene name** | **Acronym** | **Accession number** | **Primers** | **Primer location** | **TM (°C)** | **Amplicon Length (bp)** | **Reference** |
| --- | --- | --- | --- | --- | --- | --- | --- |
|  | ***Target genes*** | | | | | | |
| *Acetyl-CoA Carboxylase Alpha* | *ACACA* | NM_001114269.1 | F: 5’-AAATCCACAATGCCAACCC-3’ | Exon 54 | 66 | 87 |  |
|  |  |  | R: 5’-TCACTGTTCCTTCCACCTCC-3’ | Exon 54 |  |  |  |
| *Adiponectin, C1Q And Collagen*  *Domain Containing* | *ADIPOQ* | NM_214370.1 | F: 5’-ACGACCAGTACCAGGACA-3’ | Exon 3 | 60 | 101 |  |
|  |  |  | R: 5’-TCTCTTCATCCCCGTATG-3’ | Exon 3 |  |  |  |
| *ATP Citrate Lyase* | *ACLY* | NM_001257276.1 | F: 5’-CCTTATCCTGAATGTAGACGGTTT-3’ | Exon 27 | 60 | 151 | [1] |
|  |  |  | R: 5’-AATGAAGCCCATACTCCTTCC-3’ | Exon 28 |  |  |  |
| *Adiponectin Receptor protein1* | *ADIPOR1* | NM_001007193.1 | F: 5’-GCTGAAGGACAACGACTA-3’ | Exon 15 | 66 | 100 |  |
|  |  |  | R: 5’-GCCAGTCTCTGTGTGGAT-3’ | Exon 15 |  |  |  |
| *Adiponectin Receptor protein2* | *ADIPOR2* | NM_001007192.1 | F: 5’-TTGTAAGGTGTGGGAAGGTC-3’ | Exon 3-4 | 66 | 156 |  |
|  |  |  | R: 5’-TGCCCGTCTCTGTGTGTATT-3’ | Exon 4 |  |  |  |
| *Adipose Triglyceride Lipase* | *ATGL* | NM_001098605.1 | F: 5’-CTTCATTCCCGTGTACTGCG-3’ | Exon 3 | 66 | 93 |  |
|  |  |  | R: 5’-TCGTAGAGAGGCAGGTTGTC-3’ | Exon 4 |  |  |  |
| *Carbohydrate-responsive*  *element-binding protein* | *CHREBP* | XM_021086329.1 | F: 5’-GCCGTCATCTTGGAGGGGAA-3’ | Exon 4 | 66 | 93 |  |
|  |  |  | R: 5’-CGGAGCCGCTTCTTGTAGTA-3’ | Exon 4-5 |  |  |  |
| *Elongation Of Long Chain*  *Fatty Acids* | *ELOVL6* | XM_021100707.1 | F: 5’-AGCAGTTCAACGAGAACGAAGCC-3’ | Exon 1 | 66 | 103 |  |
|  |  |  | R: 5’-TGCCGACCGCCAAAGATAAAG-3’ | Exon 2 |  |  |  |
| *Fatty Acid Desaturase 2* | *FADS2* | NM_001171750.1 | F: 5’-CCTTCATCCTTGCTACCT-3’ | Exon 3 | 66 | 102 |  |
|  |  |  | R: 5’-CTTGTGGACGATGTGGTT-3’ | Exon 4 |  |  |  |
| *Fatty Acid Synthase* | *FASN* | NM_001099930.1 | F: 5’-ATGCCGAAGGGACCGGCTAT-3’ | Exon 4-5 | 67 | 93 | [2] |
|  |  |  | R: 5’-CATTGAGGATGGTGGCGTAT-3’ | Exon 5 |  |  |  |
| *Glucose-6-Phosphate Dehydrogenase* | *G6PD* | XM_003360515.5 | F: 5’-ACCGAGACAACATCGCCT-3’ | Exon 6 | 63 | 110 |  |
|  |  |  | R: 5’-ATTCTGCATCACGTCCCG-3’ | Exon 6-7 |  |  |  |
| *Lipase E* | *LIPE* | NM_214315.3 | F: 5’-AAGTCTACAGTGTGAGGGCC-3’ | Exon 9 | 66 | 96 | [2] |
|  |  |  | R: 5’-CGATGGGAGCTGAGTAGAGG-3’ | Exon 9 |  |  |  |
| *Lipoprotein Lipase* | *LPL* | NM_214286.1 | F: 5’-CTGCTCCTAGTGGCTCTGAG-3’ | Exon 1 | 67 | 85 | [2] |
|  |  |  | R: 5’-CTCCTGAAATTCTGTCGGCG-3’ | Exon 1-2 |  |  |  |
| *Liver X Receptor-Alpha* | *LXRA* | NM_001101814.1 | F: 5’-GCTGGGGAATGAGCTGTG-3’ | Exon 4 | 66 | 105 |  |
|  |  |  | R: 5’-TGATGACACTGCGACGGA-3’ | Exon 4 |  |  |  |
| *Malic Enzyme 1* | *ME1* | XM_001924333.5 | F: 5’-TCAAGGCTGTCGTGGTGA-3’ | Exon 4-5 | 66 | 115 |  |
|  |  |  | R: 5’-ACCCCTCCACATGCTGTA-3’ | Exon 5 |  |  |  |
| *Monoglyceride Lipase* | *MGLL* | NM_001143718.1 | F: 5’-CCACAGAGTGTCCCATACCA-3’ | Exon 2 | 66 | 95 |  |
|  |  |  | R: 5’-CTGGGTGTAGCTGAGGGTTT-3’ | Exon 2 |  |  |  |
| *Perilipin 2* | *PLIN2* | NM_214200.2 | F: 5’-ATGCTAAAGGGGCTGTGACT-3’ | Exon 5 | 67 | 103 |  |
|  |  |  | R: 5’-GTCTTGTCCACCACCTCAGT-3’ | Exon 5 |  |  |  |
| *Perilipin 3* | *PLIN3* | NM_001031778.1 | F: 5’-CGTGGTGGATTATGTGGCTC-3’ | Exon 8 | 67 | 88 |  |
|  |  |  | R: 5’-CTCCTTCTGGGGGCTTTCTCCA-3’ | Exon 8 |  |  |  |
| *Perilipin 5* | *PLIN5* | NM_001123135.1 | F: 5’-GTGGAGCTCAAACGATCCAT-3’ | Exon 5 | 60 | 102 | [3] |
|  |  |  | R: 5’-TCAGTCATGGGCAGGAAGT-3’ | Exon 5 |  |  |  |
| *Peroxisome Proliferator Activated*  *Receptor Alpha* | *PPARA* | NM_001044526.1 | F: 5’-CTTGGACTTGAACGACCAGG-3’ | Exon 6 | 60 | 96 |  |
|  |  |  | R: 5’-TCCCGTCCTTGTTCATCACA-3’ | Exon 6 |  |  |  |
| *Protein Phosphatase 3 Catalytic*  *Subunit Alpha* | *PPP3CA* | NM_214128.1 | F: 5’-ATGGGAAACCTCGTGTGGAT-3’ | Exon 2 | 60 | 100 |  |
|  |  |  | R: 5’- ATTGAAGCCCCCTCTGTTA -3’ | Exon 2 |  |  |  |
| *Retinoid X Receptor Alpha* | *RXRA* | XM_021071642.1 | F: 5’-CGAGCCCAAGACCGAGACG-3’ | Exon 6 | 66 | 166 |  |
|  |  |  | R: 5’-ACCAGGGTGAAGAGCTGCT-3’ | Exon 7 |  |  |  |
| *Stearoyl-CoA Desaturase* | *SCD* | NM_213781.1 | F: 5’-CCGGGAGAATATCCTGGTTT-3’ | Exon 5 | 66 | 56 | [2] |
|  |  |  | R: 5’-GGTAGTTGTGGAAGCCCTCA-3’ | Exon 6 |  |  |  |
| *Sterol Regulatory Element Binding Transcription Factor 1* | *SREBP1C* | NM_214157.1 | F: 5’-CACCGTTTCTTCGTGGATGG-3’ | Exon 12 | 66 | 114 |  |
|  |  |  | R: 5’-ACGGAACAACTGAGTCACCT-3’ | Exon 13 |  |  |  |
|  | ***Housekeeping genes*** | | | | | | |
| *Actin Beta* | *ACTB* | XM_021086047.1 | F: 5’-CCAGGTCATCACCATCGG-3’ | Exon 4 | 66 | 158 |  |
|  |  |  | R: 5’-CCGTGTTGGCGTAGAGGT-3’ | Exon 5 |  |  |  |
| *Beta-2-Microglobulin* | *B2M* | NM_213978.1 | F: 5’-CCTTCTGGTCCACACTGAGT-3’ | Exon 2 | 66 | 99 | [2] |
|  |  |  | R: 5’-TCCCACTTAACTATCTTGGGCT-3’ | Exon 2-3 |  |  |  |
| *Hypoxanthine Phosphoribosyltransferase 1* | *HPRT1* | NM_001032376.2 | F: 5’-CCCAGCGTCGTGATTAGTGA-3’ | Exon 1-2 | 66 | 88 | [2] |
|  |  |  | R: 5’-CCTTTTCCAAATCCTCGGCA-3’ | Exon 2 |  |  |  |
| *RNA Polymerase II Subunit A* | *POLR2A* | XM_021067825.1 | F: 5’-GGGACTCCATTGCTGATTCT-3’ | Exon 13 | 66 | 92 | [2] |
|  |  |  | R: 5’-GCCTTCTCGATGACCTC-3’ | Exon 13-14 |  |  |  |
| *Ribosomal Protein Lateral Stalk Subunit P0* | *RPLP0* | NM_001098598.1 | F: 5’-AGACAAAGTGGGAGCCAGTG-3’ | Exon 5 | 63 | 111 |  |
|  |  |  | R: 5’- GGGTTGTAGATGCTGCCATT-3’ | Exon 5 |  |  |  |
| *Ribosomal Protein L32* | *RPL32* | NM_001001636.1 | F: 5’-TCATGGCTGCTCTCAGACCC-3’ | Exon 2 | 63 | 145 |  |
|  |  |  | R: 5’-CTTCTCCGCACCCTGTTGT-3’ | Exon 3 |  |  |  |
| *Ribosomal Protein S18* | *RPS18* | NM_213940.1 | F: 5’-CATGTGGTGTTGAGGAAAGCA-3’ | Exon 3 | 63 | 105 | [4] |
|  |  |  | R: 5’-TTGGCGAGGATTCTGCATAAT-3’ | Exon 4 |  |  |  |
| *TATA-Box Binding Protein* | *TBP* | XM_021085497.1 | F: 5’-GATGGACGTTCGGTTTAGG-3’ | Exon 10 | 63 | 124 |  |
|  |  |  | R: 5’-AGCAGCACAGTACGAGCAA-3’ | Exon 10 |  |  |  |
| *Tyrosine 3-Monooxygenase/Tryptophan*  *5-Monooxygenase Activation Protein Zeta* | *YWHAZ* | NM_001315726.1 | F: 5’-AAGGCGTAGTGGAAGTGGAT-3’ | Exon 6 | 66 | 98 | [2] |
|  |  |  | R: 5’-GCTGTAGTCAAAGGTGTGCA-3’ | Exon 6 |  |  |  |

**Literature cited in supplementary material:**

1. Zappaterra M, Braglia S, Bigi M, Zambonelli P, Davoli R. Comparison of expression levels of fourteen genes involved in the lipid and energy metabolism in two pig breeds. Livest Sci. 2015;181: 156–162.

2. Zappaterra M, Deserti M, Mazza R, Braglia S, Zambonelli P, Davoli R. A gene and protein expression study on four porcine genes related to intramuscular fat deposition. Meat Sci. 2016;121: 27-32.

3. Zappaterra M, Mazzoni M, Zambonelli P, Davoli R. Investigation of the Perilipin 5 gene expression and association study of its sequence polymorphism with meat and carcass quality traits in different pig breeds. Animal. 2018;12: 1135-1143.

4. Vitali M, Conte S, Lessard M, Deschêne K, Benoit-Biancamano MO, Celeste C, et al. Use of the spectrophotometric color method for the determination of the age of skin lesions on the pig carcass and its relationship with gene expression and histological and histochemical parameters. J Anim Sci. 2017;95: 3873-3884.
