# Supplementary material for: Effect of dietary polyunsaturated fatty acid and antioxidant supplementation on the transcriptional level of genes involved in lipid and energy metabolism in swine
Source: PLoS One. 2018 Oct 4;13(10):e0204869. doi: 10.1371/journal.pone.0204869 (PMC6171869; doi:10.1371/journal.pone.0204869)
Supplement: S3 Table — P-values of the comparisons are reported on the top of each cell and the means and standard errors for each diet are shown between brackets. Legend: D1 = standard diet for growing-finishing pigs; D2 = standard diet supplemented with linseed; D3 = standard diet supplemented with linseed, vitamin E and selenium; D4 = standard diet supplemented with linseed and plant extracts from grape-skin and oregano as source of polyphenols. (DOC) [file pone.0204869.s004.doc]

| **Target gene** | **D1-D2** | **D1-D3** | **D1-D4** | **D2-D3** | **D2-D4** | **D3-D4** |
| --- | --- | --- | --- | --- | --- | --- |
| ***FASN*** | 0.792  [4.13±1.28 - 3.54±1.78] | 0.970  [4.13 ±1.28 - 4.99±1.07] | 0.134  [4.13±1.28 - 12.47±4.54] | 0.530  [3.54 ±1.78 - 4.99±1.07] | 0.020  [3.54 ±1.78 - 12.47±4.55] | 0.289  [4.99 ±1.07 - 12.47 ±4.54] |
| ***ELOVL6*** | 0.998  [0.05±0.01 - 0.06±0.01] | 0.980  [0.05±0.01 - 0.05±0.01] | 0.005  [0.05±0.009 - 0.13±0.028] | 0.997  [0.06±0.01 - 0.05±0.01] | 0.009  [0.06±0.01 - 0.13 ±0.03] | 0.013  [0.05 ±0.01 - 0.13 ±0.03] |
| ***SCD*** | 0.213  [5.25±1.52 - 2.23±0.68] | 0.932  [5.25 ±1.52 - 3.92±0.96] | 0.116  [5.25±1.52 - 11.83±3.51] | 0.508  [2.23±0.68 - 3.92±0.96] | 0.001  [2.23 ±0.68 - 11.83±3.51] | 0.033  [3.92±0.96 - 11.83±3.51] |
| ***ACACA*** | 0.098  [1.18±0.26 - 0.64±0.31] | 0.303  [1.18±0.26 - 0.59±0.11] | 0.257  [1.18±0.26 - 2.13±0.56] | 0.918  [0.64±0.31 - 0.59±0.11] | 0.001  [0.64±0.31 - 2.13±0.56] | 0.005  [0.59±0.11 - 2.13±0.56] |
| ***ADIPOQ*** | 0.727  [0.08±0.02 - 0.07±0.04] | 0.990  [0.08±0.02 - 0.06±0.01] | 0.103  [0.08±0.02 - 0.18±0.06] | 0.882  [0.07±0.04 - 0.06±0.01] | 0.011  [0.07±0.04 - 0.18±0.06] | 0.054  [0.06±0.01 - 0.18±0.06] |
| ***LXRA*** | 1.000  [2.96±0.45 - 2.82±0.34] | 0.662  [2.96±0.45 - 3.73±0.47] | 0.880  [2.96±0.45 - 3.59±0.66] | 0.609  [2.82±0.34 - 3.73±0.47] | 0.842  [2.82±0.34 - 3.59±0.66] | 0.981  [3.73±0.47 - 3.59±0.66] |
| ***CHREBP*** | 0.509  [0.006±0.001 - 0.004±0.001] | 0.999  [0.006±0.001 - 0.005±0.001] | 0.209  [0.006±0.001 - 0.008±0.002] | 0.410  [0.004±0.001 - 0.005±0.001] | 0.010  [0.004±0.001 - 0.008±0.002] | 0.272  [0.005±0.001 - 0.008±0.002] |
| ***FADS2*** | 0.802  [0.07±0.02 - 0.10±0.02] | 0.639  [0.07±0.02 - 0.12±0.03] | 0.502  [0.07±0.02 - 0.12±0.03] | 0.993  [0.10±0.02 - 0.12±0.03] | 0.957  [0.10±0.02 - 0.12±0.03] | 0.994  [0.12±0.03 - 0.12±0.03] |
| ***ADIPOR1*** | 0.0363  [9.93±2.06 - 5.02±1.31] | 0.530  [9.93±2.06 - 7.06±1.49] | 0.407  [9.93±2.06 - 13.01±2.17] | 0.448  [5.02±1.31 - 7.06±1.49] | 0.001  [5.02±1.31 - 13.01±2.17] | 0.032  [7.06±1.49 - 13.01±2.17] |
| ***ADIPOR2*** | 0.669  [0.14±0.04 - 0.08±0.01] | 0.949  [0.14±0.04 - 0.11±0.02] | 0.449  [0.14±0.04 - 0.18±0.03] | 0.929  [0.08±0.01 - 0.11±0.02] | 0.066  [0.08±0.01 - 0.18±0.03] | 0.200  [0.11±0.02 - 0.18±0.03] |
| ***PLIN3*** | 0.585  [2.42±0.61 - 2.96±0.35] | 0.261  [2.42±0.61 - 3.07±0.51] | 0.744  [2.42±0.61 - 2.42±0.31] | 0.939  [2.96±0.61 - 3.07±0.51] | 0.995  [2.96±0.61 - 2.42±0.31] | 0.847  [3.07±0.51 - 2.42±0.31] |
| ***PLIN2*** | 0.172  [1.80±0.23 - 1.24±0.14] | 0.841  [1.80±0.23 - 1.67±0.32] | 0.638  [1.80±0.23 - 2.08±0.28] | 0.561  [1.24±0.14 - 1.67±0.32] | 0.014  [1.24±0.14 - 2.08±0.28] | 0.214  [1.67±0.32 - 2.08±0.28] |
| ***ME1*** | 1.000  [0.83±0.21 - 0.75±0.12] | 0.465  [0.83±0.21 - 0.84±0.08] | 0.938  [0.83±0.21 - 0.94±0.24] | 0.470  [0.75±0.12 - 0.84±0.08] | 0.939  [0.75±0.12 - 0.939±0.24] | 0.825  [0.84±0.08- 0.939±0.24] |
| ***LIPE*** | 1.000  [0.63±0.14 - 0.64±0.14] | 0.870  [0.63±0.14 - 0.63±0.07] | 0.669  [0.63±0.14 - 0.92±0.32] | 0.843  [0.64±0.14 - 0.63±0.07] | 0.637  [0.64±0.14 - 0.92±0.32] | 0.979  [0.63±0.07 - 0.92±0.32] |
| ***G6PD*** | 0.167  [0.12±0.03 - 0.27±0.05] | 0.043  [0.12±0.03 - 0.27±0.06] | 0.025  [0.12±0.03 - 0.31±0.60] | 0.938  [0.27±0.05 - 0.27 ±0.06] | 0.826  [0.27±0.05 - 0.31±0.60] | 0.991  [0.27±0.062 - 0.31±0.60] |
| ***LPL*** | 0.954  [0.19±0.06 - 0.20±0.03] | 0.771  [0.19±0.06 - 0.20±0.03] | 0.266  [0.19±0.06 - 0.25±0.04] | 0.973  [0.20±0.03 - 0.20±0.03] | 0.552  [0.20±0.03 - 0.25±0.04] | 0.797  [0.20±0.03 - 0.25±0.04] |
| ***RXR*** | 0.172  [0.11±0.03 - 0.05±0.01] | 0.982  [0.11±0.03 - 0.08±0.01] | 0.679  [0.11±0.03 - 0.15±0.03] | 0.285  [0.05± 0.01 - 0.08±0.01] | 0.014  [0.05± 0.01 - 0.15±0.03] | 0.463  [0.08±0.01 - 0.15±0.03] |
| ***SREBP1C*** | 0.998  [0.04±0.01 - 0.05±0.01] | 0.696  [0.04±0.01 - 0.05±0.01] | 0.823  [0.04± 0.01 - 0.06±0.01] | 0.805  [0.05±0.01 - 0.05±0.01] | 0.906  [0.05±0.01 - 0.06±0.01] | 0.997  [0.05±0.01 - 0.06±0.01] |
| ***PPP3CA*** | 1.000  [11.22±2.61 - 9.91±1.07] | 0.997  [11.22± 2.61 - 10.94± 1.91] | 0.962  [11.22±2.61 - 12.34±2.27] | 0.998  [9.91±1.07 - 10.94±1.91] | 0.968  [9.91±1.07 - 12.34±2.27] | 0.991  [10.94±1.91 - 12.34±2.27] |
| ***PPARA*** | 0.461  [0.05±0.01 - 0.04±0.01] | 0.901  [0.05±0.01 - 0.04±0.01] | 0.752  [0.05±0.01 - 0.06±0.01] | 0.844  [0.04±0.01 - 0.04±0.01] | 0.088  [0.04±0.01 - 0.06±0.01] | 0.361  [0.04±0.01 - 0.06±0.01] |
| ***ATGL*** | 0.942  [0.05±0.01 - 0.04±0.01] | 0.715  [0.05±0.01 - 0.04±0.01] | 0.909  [0.05±0.01 - 0.06±0.01] | 0.963  [0.04±0.01 - 0.04±0.01] | 0.622  [0.04±0.01 - 0.06±0.01] | 0.342  [0.04±0.01 - 0.06±0.01] |
| ***ACLY*** | 0.798  [0.47±0.14 - 0.54±0.10] | 0.995  [0.47±0.14 - 0.36±0.06] | 0.929  [0.47±0.014 - 0.49±0.13] | 0.660  [0.54±0.10 - 0.36±0.06] | 0.991  [0.54±0.10 -0.49±0.13] | 0.832  [0.36±0.06 - 0.49±0.13] |
| ***PLIN5*** | 0.999  [0.68±0.19 - 0.86±0.23] | 0.591  [0.68±0.19 - 0.51 ±0.25] | 1.000  [0.68 ±0.19 - 0.72±0.28] | 0.526  [0.86±0.23 - 0.51±0.25] | 0.997  [0.86±0.233 - 0.72±0.280] | 0.662  [0.51±0.25 - 0.72±0.28] |
| ***MGLL*** | 0.968  [0.11±0.02 - 0.08±0.01] | 0.955  [0.11±0.02 - 0.13±0.05] | 0.680  [0.11±0.02 - 0.21±0.07] | 1.000  [0.08±0.01 - 0.13±0.05] | 0.424  [0.08±0.01 - 0.21±0.07] | 0.382  [0.13±0.05 - 0.21±0.07] |
